# Energy consumption analysis and optimization in collaborative robots

**DOI:** 10.3389/frobt.2025.1671336

**Published:** 2025-10-31

**Authors:** Sofia Miranda, Carlos Renato Vázquez, Manuel Navarro-Gutiérrez

**Affiliations:** 1 Departamento de Transformación Digital, Aumovio, Tlajomulco de Zuñiga, Mexico; 2 Escuela de Ingenieria y Ciencias, Tecnologico de Monterrey, Zapopan, Mexico

**Keywords:** sustainable manufacturing, smart manufacturing, industry 4.0, collaborative robot, energy consumption

## Abstract

Energy consumption is a key concern in modern industrial facilities. Power peak is also a relevant feature in industrial energy analysis and managment, since the electrical infrastructure must be implemented to provide not only the total consumed energy, but the power peaks. Collaborative robots are gaining popularity due to its flexible use and convenient set up. In this context, a power and energy consumption study of the popular UR10 collaborative robot of Universal Robots is reported in this work. For this, an experiment was conducted to obtain current consumption data from the UR10 API, when performing movements with different loads and parameters. Next, the dependency of the trajectory programming parameters on the power peak, total consumed energy, and time spent per trajectory was analyzed. The results show that the higher the speed limit and acceleration limit, the lower the total energy consumed per trajectory, but the higher the power peak. This behavior represents a trade-off: reducing the consumed energy involves increasing the peak power. Based on the captured data, artificial neural network models were trained to predict the power peak and the total energy consumed by the robot when performing a movement under certain parameters. These models were later used by a genetic optimization algorithm to obtain the best parameters for a given target position, providing the most efficient performance while fulfilling a power peak bound.

## 1 Introduction

The use of smart manufacturing concepts and technologies is becoming fundamental for improving productivity, driven by the rapid rise of automation in fast-paced mass manufacturing environments. This growth is reflected in the number of installed robots during the last years. According to the International Federation of Robotics (IFR), the operational stock of industrial robots has been increasing by 12% each year since 2018. Starting in 2025, growth is projected at an average annual rate of 4%. This trend requires the search for strategies to optimize the resources needed for their operation. In the same way, the IFR states that human-robot collaboration continues developing and expanding in different applications.

Collaborative robotics has brought new opportunities to factory automation. Nowadays, cobots offer an economically feasible way to begin robotic automation, in contrast to traditional industrial robots, which often require larger investments, dedicated infrastructure, and specialized personnel. Collaborative robots can be used to support workers in complex assembly tasks, they are capable of detecting collisions and automatically stopping for preventing robot and operators damage, they have intuitive programming interfaces that allow workers with minimal training to re-deploy robots to new tasks, they can be easily relocated within the factory due to their lightweight and compact design (International Federation of Robotics, 2021), they incorporate sensors that allow control force, they require fewer safety devices, which helps reduce overall costs for manufacturers, among others.

As pointed out by the IFR, a rising concern for the future of robotics is the sustainability, driving manufacturing companies to seek more efficient ways to utilize robots. Energy consumption in robotics is a critical resource that needs to be optimized, as it significantly impacts the return of investment, and is part of the sustainable indicators of the complete manufacturing process. Moreover, the complexity of robotic solutions is increasing due to the new functionalities of modern robotic manufacturing systems, the growing production demands, and the rise of customized mass production (Schäffer et al., 2019). As a result, robotic programs must be carefully optimized to ensure efficiency and adaptability. In this context, the open communication architecture of collaborative robots can contribute to greater efficiency by enabling the collection and sharing of data from their sensors, which facilitates the improvement and optimization of their operation.

The analysis of energy consumption in robotics has received attention in the literature in recent years. In Gadaleta et al. (2019); Pellicciari et al. (2013), the optimization of motion parameters of an industrial (traditional) robot for efficient energy use was addressed. In these studies, an algorithm for an efficient use of energy, by correctly adjusting the maximum speed and acceleration, was presented. Figure 1b in Pellicciari et al. (2013) shows the energy consumption versus the time required to perform a task: it can be seen that a too slow and a too fast execution (corresponding to a large and a small task execution time, respectively) requires more energy; the most efficient execution is performed at a certain medium speed ( $1/T_{\mathrm{opt}}$ in Figure 1b in Pellicciari et al. (2013)). Guerra-Zubiaga and Luong (2021) analyzed the energy consumption of an industrial (traditional) robot using a linear factorial experiment analysis, its goal was to determine the parameters that contribute most to the robot energy consumption. Carabin et al. (2017); Soori et al. (2023) reviewed energy-saving optimization methodologies for robotic systems, including works that explore saving energy by optimizing robot trajectories and performing operation scheduling. For instance, De Laet et al. (2025) proposed a motion profile optimization based on Chebyshev polynomials, so the energy consumption through a trajectory is minimized while the trajectory time is maintained. Hou et al. (2021) studied the energy consumption on mobile robots, focusing on motion planning and task planning by using a Matlab simulator. Nonoyama et al. (2022) researched on the energy optimization for optimal motion planning for an industrial robot, by tuning a PID controller using a Genetic Algorithm. There is special interest in the literature for energy consumption prediction: Jiang et al. (2023) proposed and LSTM neural network model for the energy prediction of an industrial robot, considering a time scaling function as a parameter to be optimized; in Wang et al. (2025b), a model architecture that predicts the joints torques and power losses in a separated way are applied, these variables are later used to calculate the energy consumption; in Wang et al. (2025a), a different architecture is considered, and transfer learning is proposed as a mechanism for adapting the model to a different robot. Aliev et al. (2021) proposed a prediction and estimation model for energy demand of a mobile robot and a cobot by using linear regression, its objective was to evaluate the battery behavior during the robots task execution.

Some works have recently considered the energy consumption in collaborative robots (cobots). In Boscariol et al. (2023), a framework for studying the energy consumption of a cobot UR5e is presented, which consists in a dynamic model based on physics whose parameters are tuned with data obtained from real experiments. A similar model is implemented in a digital twin of a cobot UR3e in Heredia et al. (2023) for training purposes, so the user can try different programs that result in better power consumptions. The authors argue that the power estimation can be used to detect anomalies in the real robot. A Deep Deterministic Policy Gradient scheme is used in Gorkavyy et al. (2025) for the trajectory optimization in collaborative robots. In Vodovozov and Raud (2025), a data-driven procedure for robot tool location and parameters optimization is presented.

Nevertheless, despite the large amount of studies addressing the energy consumption of industrial robots, the power peak has not been properly studied. Power peak is a relevant feature in industrial energy analysis and managment, since the electrical infrastructure must be implemented to provide not only the total consumed energy, but the power peaks. Moreover, power peak affects the energy costs. Energy managers may implement strategies for reducing power peak demands in the facilities, a concept know as peak load shaving, which may include the limitation or peak power requirements at certain hours Silva et al. (2020).

Inspired by the referenced works on energy consumption in industrial robots and mobile robots, this work analyzes the power and energy consumption of a collaborative robot UR10 from Universal Robots, one of the most popular cobot in the market, as a function of the motion parameters (load, initial and target coordinates, speed limit, acceleration limit, and movement type). For this, an experimental setting is implemented with the cobot, taking data from the robot sensors via the manufacturer’s API (a key difference from the works dealing with industrial robots, where data is usually obtained from simulations since those robots do not provide sensors data). The analysis performed shows that the larger the speed limit of the collaborative robot movement, the lower the total energy consumption in the movement, obtaining a relation between execution time and energy consumption different from that of industrial robots (Figure 1b in Pellicciari et al. (2013)). On the other hand, increasing the speed limit causes the increase of the power peak. The experimental data, obtained from 1,450 performed trajectories, is later used to train a couple of artificial neural networks (ANN) to predict the energy consumption during a movement, as a function of the movement parameters, and the power peak. Finally, these models are used by an optimization genetic algorithm to obtain the parameters (speed limit and acceleration limit) that provide the lowest energy consumption while keeping the power peak inside a required bound, given the initial and targe coordinates and payload. The difference of this study in comparison with those of the literature relies in four aspects: 1) here a collaborative robot is analyzed rather than an industrial (traditional) robot, 2) a real robot is used (no simulators) without additional sensors or equipment, the data used is that already provided by the robot (allowing to replicate and scale the predictor algorithms), 3) the power peak is also analyzed, since this variable is relevant for energy planning, 4) and optimization algorithm is introduced for obtaining the parameters that provide the minimum energy consumption while keeping the power peak inside a required bound. Trajectory planning is not considered here, since a practical optimization in industrial environments should only involve the adjustment of the motion parameters in the cobot controller, avoiding the modification of the controller path planning algorithm.

Some preliminary results were presented in the conference paper Miranda and Vázquez (2023), where the energy consumption analysis and the ANN predictor were reported, without addressing the power peak analysis and prediction, and the energy optimization.

The organization of the manuscript is as follows. The experimental setting and methodology are described in Section 2. The results obtained from the experiments are reported in Section 3. A discussion on these results, describing the relation between the movement parameters and the energy consumption, power peak and trajectory time, is presented in Section 4. The training of energy consumption and peak power predictors are reported in Section 5. The use of these predictors for calculating the optimal speed and acceleration limits is described in Section 6. Finally, some conclusions are presented in Section 7.

## 2 Materials and methods

In this section, the experimental setting and the three performed experiments are described.

### 2.1 Hardware description

This work focuses on a collaborative robot from Universal Robots, the UR10 model, which is among the most widely used in the market. The UR10 is a 6-axis articulated robot with a maximum payload capacity of 10kg and a reach of up to 1300mm from its base axis. The robot itself has a total mass of 28.9 kg .

The maximum velocity of each axis is defined by the technical specifications of the motors and gear mechanisms. In the case of the UR10 robot, the maximum velocity is 120°/s for axes 1 and 2, and 180°/s for axes 3 through 6. Each axis has current and voltage sensors, which makes possible to compute the energy consumption in every axis of the robot.

In order to store the data generated by the robot during operation, a PC is used. The transfer of the data is done through a TCP/IP protocol, establishing the communication between the PC and the robot controller via the Real-Time Data Exchanger (RTDE) interface. The output messages are generated at a frequency of 125Hz . The extracted data are then stored in a. csv file for subsequent offline analysis.

In particular, the DC current and voltage consumed by the robot actuators were recorded during its operation. These measurements enabled the calculation of the instantaneous real power at each time, which was then numerically integrated over time to determine the total energy consumption for a single trial (i.e., the execution of one trajectory).

### 2.2 Experiment 1

In the first experiment, the robot performs vertical movements to analyze the influence of movement parameters (such as speed and acceleration limits) on the energy consumption and power peak.

For this experiment, the robot’s tool center point (TCP) was moved exclusively along the z-axis, without displacements in other directions. The motion followed a straight-line path using a linear movement command (MoveL). All test runs in this experiment followed the same trajectory, covering a distance of 800mm .

The maximum speed of the TCP was varied between 0.1m/s to 0.9m/s . In the case of acceleration, it was set to $1.6\;m/s^{2}$ or $3.5\;m/s^{2}$ . Additionally, during different test runs, the robot carried loads of 0kg , 2.58kg , and 5kg . Table 1 briefly presents the test runs of this experiment.

**TABLE 1:** Experiment 1. In all the trials, the movement was of type MoveL, and the traveled distance was 800mm in axis Z.

Trial	v (m/s)	a $\left(m/s^{2}\right)$	d (mm)	Load (kg)
1	0.1–0.9	1.6	800	0
2	0.1–0.9	1.6	800	2.58
3	0.1–0.9	1.6	800	5
4	0.5–0.9	1.6	800	0
5	0.5–0.9	1.6	800	2.58
6	0.5–0.9	1.6	800	5
7	0.1–0.9	3.5	800	0
8	0.1–0.9	3.5	800	2.58
9	0.1–0.9	3.5	800	5
10	0.5–0.9	3.5	800	0
11	0.5–0.9	3.5	800	2.58
12	0.5–0.9	3.5	800	5

### 2.3 Experiment 2

For the second experiment, the robot performs horizontal and vertical movements using the commands MoveL and MoveJ to study the influence of the movement parameters on the consumed energy and power peak.

In this experiment, the robot’s TCP was programmed to move through four distinct positions involving both horizontal and vertical displacements, specifically along the X and Z axes. All test runs in this experiment followed the same trajectory, in which the robot’s TCP reaches the corners of a square. The trajectory begins with a displacement of 400mm along the X-axis, followed by a second move of 400mm along the Z-axis. The third move involves a return of 400mm in the negative direction of the X-axis, and the TCP returns to its starting point with a 400mm displacement in the negative Z direction. For achieving this trajectory, some test runs were performed using a movement of type MoveL, and another ones were done using a movement of type MoveJ.

The maximum speed of the TCP was varied between 0.5m/s and 0.9m/s , while the acceleration was kept constant at $3.5\;m/s^{2}$ . For different test runs, the robot carried loads of 0kg , 2.58kg and 5kg . Table 2 summarizes the trials conducted in this experiment.

**TABLE 2:** Experiment 2. In all the trials, the acceleration limit was set to $3.5\;m/s^{2}$ , and the displacement was 400mm in axis X and 400mm in axis Z.

Trial	Type	v (m/s)	Load (kg)
1	MoveL	0.5–0.9	0
2	MoveL	0.5–0.9	2.58
3	MoveL	0.5–0.9	5
4	MoveJ	0.5–0.9	0
5	MoveJ	0.5–0.9	2.58
6	MoveJ	0.5–0.9	5

### 2.4 Experiment 3

**FIGURE 1:**
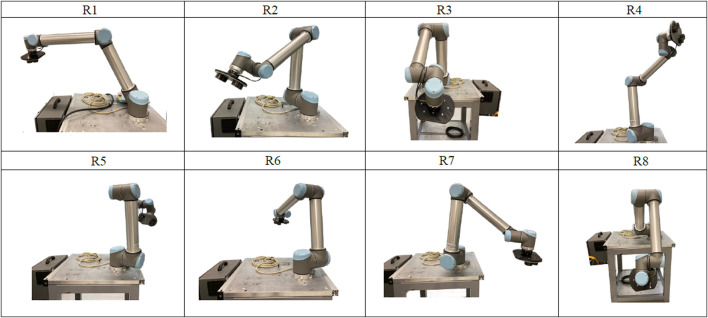
Positions of the UR10 robot, described in Table 3, for Experiment 3.

For the third experiment, the robot follows a trajectory composed by eight points that were randomly generated. These points are listed in Table 3 and the corresponding positions are shown in Figure 1. The robot’s TCP moves sequentially through these points, starting at P1 , continuing through P8 , and finally returning to P1 to complete the loop.

**TABLE 3:** Positions for Experiment 3. The X, Y and Z coordinates are expressed in m , RX, RY and RZ are expressed in rad .

P	X	Y	Z	RX	RY	RZ
1	−0.6303	−0.7869	0.5295	−0.7995	−2.7028	−0.0962
2	−0.0275	−0.6599	−0.6599	−0.4853	2.9140	−0.8602
3	0.7200	−0.4777	0.2224	1.6754	−2.2398	−0.7478
4	0.4855	−0.0305	1.1396	−1.4307	0.8210	−0.9978
5	−0.1410	0.4735	0.5214	2.1715	0.8002	0.1797
6	−0.7575	0.1527	0.4897	−1.9442	−2.2191	0.3603
7	0.5637	0.3216	0.2721	2.9271	−1.0571	−0.1375
8	0.6165	−0.4870	−0.1024	1.3697	−2.0370	−1.2442

In this experiment, the speed limit, acceleration limit and load were varied, according to Table 4.

**TABLE 4:** Experiment 3.

Trial	Type	v (m/s)	a $\left(m/s^{2}\right)$	Load (kg)
1	MoveL	0.1–0.7	1.4–2.2	0
2	MoveL	0.1–0.7	1.4–2.2	2.58
3	MoveL	0.1–0.7	1.4–2.2	5

Trial	Type	ω (rad/s)	a $\left(rad/s^{2}\right)$	Load (kg)
4	MoveJ	1–4.2	1–7	0
5	MoveJ	1–4.2	1–7	2.58
6	MoveJ	1–4.2	1–7	5

## 3 Results

### 3.1 Experiment 1

**FIGURE 2:**
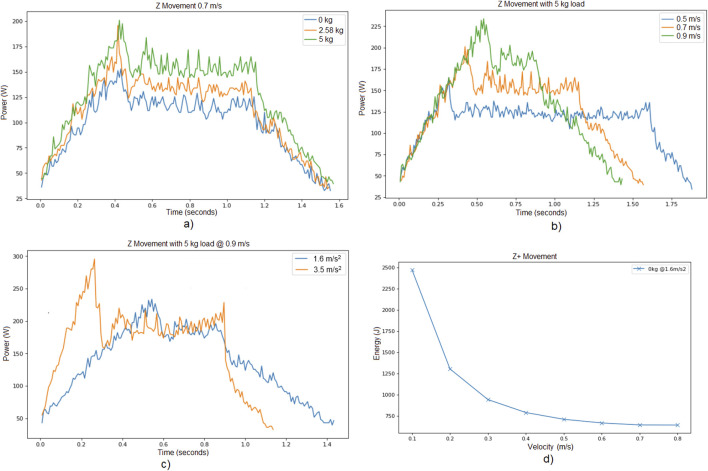
Experiment 1. Power consumed while moving along Z axis for the cases, **(a)** different payloads with the same speed and acceleration limits, **(b)** different speed limits with the same payload and acceleration limit, **(c)** different acceleration limits with the same payload and speed limit, **(d)** Energy consumed with different speed limits moving along Z axis with payload 0kg and acceleration limit $1.6\;m/s^{2}$ .

Figure 2 presents the power consumption results for various trials conducted in Experiment 1. To be more specific:

• Figure 2a illustrates the power consumed during the vertical movement phase (from the lower to the upper position) for different payloads, while maintaining a constant speed limit and acceleration limit. As expected, an increase in payload results in higher power consumption. For instance, Table 5 reports the power peak and trajectory time for the specific case of speed limit 0.7  m/s and acceleration limit $1.6\;m/s^{2}$ (trajectory times are similar, since they depend on the speed and acceleration limit parameters, rather than the load).

• Figure 2b displays the power consumption for different limit speeds, keeping both the payload and acceleration constant. As anticipated, higher speeds lead to greater power peaks. Similarly, the trajectory time becomes lower for a larger speed limit.

• Figure 2c shows the effect of varying acceleration on power consumption, with a fixed payload and speed limit. As expected, higher acceleration values result in higher peak power usage and lower trajectory times.

**TABLE 5:** Results of Experiment 1 for a speed limit 0.7  m/s and acceleration limit $1.6\;m/s^{2}$ .

Load	Power peak (W)	Trajectory time (s)
0	152.96	1.54
2.58	195.93	1.54
5	201.09	1.56

Figure 2d depicts the power consumed for different trials regarding Experiment 1. The payload was kept at 0kg for all trials, and the acceleration remained constant. The only parameter that was varied was the limit speed. Interestingly, the results show that higher limit speeds lead to lower total energy consumption. This may appear counter-intuitive, as Figure 2b shows that higher speeds lead to greater power peak consumption. However, operating at a lower speed causes the robot to take more time to complete the trajectory, which in turn leads to greater total energy consumption. In other words, when following the same trajectory, a higher limit speed results in a shorter execution time, thereby enabling a more energy-efficient execution per trajectory.

**FIGURE 3:**
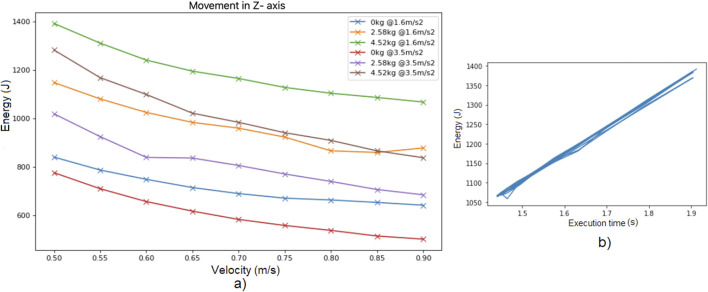
**(a)** Energy consumed while moving along Z axis for different speed limits, for different combinations of payload and acceleration limit. **(b)** Energy consumption per trajectory vs. execution time, for trials of Experiment 1 with different speed limits and accelerations but the same load of 5kg .

Finally, Figure 3a illustrates the energy consumption in Experiment 1 as the speed limit is varied, considering different combinations of acceleration limit and payload. It is interesting to note that for every payload and acceleration limit, by increasing the speed limit the total energy consumed is decreased, concluding that neither the acceleration limit nor the payload affect the observation of Figure 2d.

Figure 3b shows the energy consumption per trajectory versus the total execution time per trajectory, for trials of Experiment 1 with different speed limits and acceleration limits but the same load of 5kg . It can be observed that the relation between energy consumption and execution time is almost linear, being far different from the behavior observed in industrial (traditional) robots (Figure 1b in Pellicciari et al. (2013)).

### 3.2 Experiment 2

**FIGURE 4:**
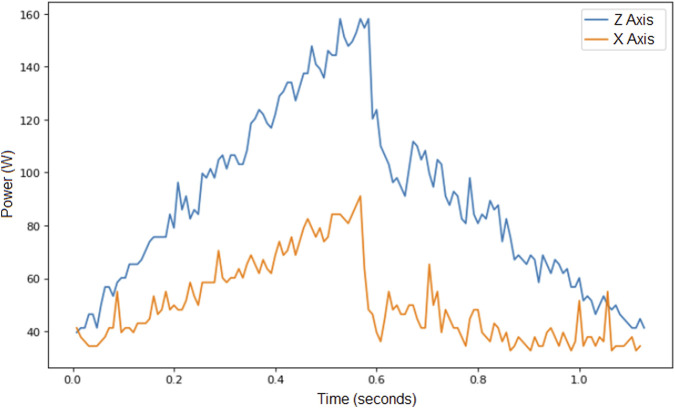
Power consumed for two movements, one along axis X and the other along axis Z, for the same speed limit, acceleration limit and payload. These trial were obtained from Experiment 2.

Regarding the data obtained from Experiment 2, Figure 4 illustrates the power consumption when the robot reaches the points of the square described above using two different movements: one along the X-axis and the other along the Z-axis. Both movements were performed under the same conditions of speed, acceleration, and payload. As expected, movement along the vertical Z-axis requires more power than movement along the X-axis, even when displacement, speed, acceleration, and payload are identical in both cases. The results from Experiment 2 consistently showed this pattern: while the influence of the parameters on power consumption is similar for movements along both axes, the overall power consumption is noticeably higher when the robot moves vertically along the Z-axis.

## 4 Discussion from experiments 1 and 2

The findings of the obtained results from experiment 1 and 2 are the following.

• Movement along Z axis consumes the most of energy. A path optimization should focus on the reduction of the movement on axis Z.

• For any given constant load and acceleration limit: increasing the speed limit increases the power peak, but decreases the trajectory time and the consumed energy per trajectory.

• For any given constant load and speed limit: increasing the acceleration limit increases the power peak, but decreases the trajectory time and the consumed energy per trajectory.

• The resulting relation between execution time and energy consumption per trajectory for a cobot (Figure 3b) is different from that of traditional industrial robots (Figure 1b in Pellicciari et al. (2013)). In particular, the cobot behavior seems to lie in the slow motion zone (right zone of Figure 1b in Pellicciari et al. (2013)), since cobots are slow in any case, thus the power required to accelerate the cobot is always small in comparison with the power required to compensate the gravity force.

• Consequently, for any given constant load, the fastest and most efficient trajectory (in terms of total energy consumed) is obtained by maximizing both the acceleration limit and the speed limit, inside the allowed ranges. Nevertheless, maximizing speed and acceleration maximizes power peak as well, thus, if there are power-peak constraints, the optimal parameters should be obtained through a deeper analysis.

The reported results were obtained with linear movements (MoveL), but similar findings were obtained for joint movements (MoveJ). However, it is important to mention that the power consumed with MoveL and MoveJ are different, for the same payload, speed limit, and acceleration limit. This is explored with more detail in the following section.

## 5 Energy and power peak predictors

The initial steps involves identifying the variables that are most strongly correlated with the energy consumption and power peak from the 32 variables obtained from the UR robotic arm.

**FIGURE 5:**
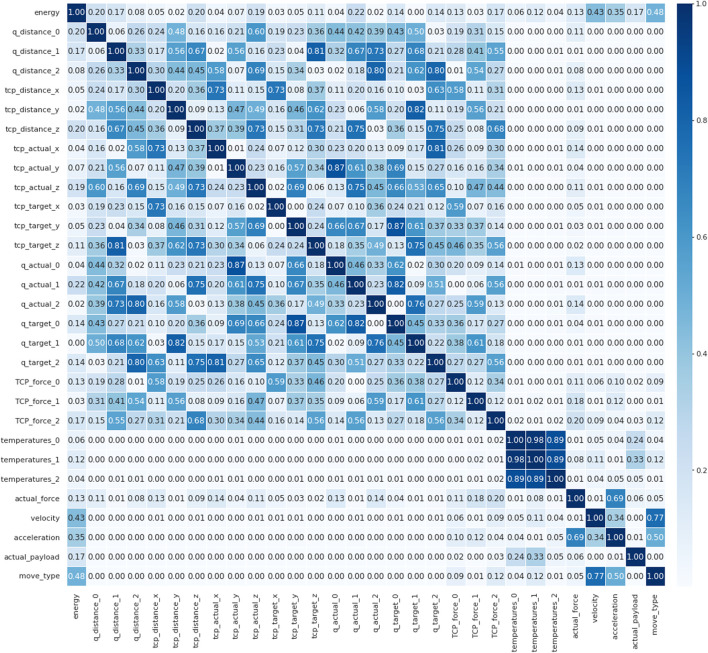
Pearson matrix. The most relevant input variables (correlation coefficient >0.12 ) are: Q_distance_0, Q_distance_1, TCP_distance_x, TCP_distance_z, TCP_actual_z, TCP_target_z, Q_actual_1, Q_target_0, Q_target_2, TCP_force_0, TCP_force_2, Actual_force, Payload, Move_type, Velocity, Acceleration.

For this, a Pearson correlation matrix was constructed, as shown in Figure 5. The matrix describes the correlation between inputs and the energy consumption. The most relevant input variables were: Q_distance_0, Q_distance_1, TCP_distance_x, TCP_distance_z, TCP_actual_z, TCP_target_z, Q_actual_1, Q_target_0, Q_target_2, TCP_force_0, TCP_force_2, Actual_force, Payload, Move_type, Velocity, and Acceleration. However, some of these variables are redundant, such as initial coordinates, target coordinates and distance (difference between initial and target coordinates). By removing some of these, the variables that were selected as the input variables to train the ANN model were the initial coordinates (x,y,z), the target coordinates (x,y,z), payload, movement type, limit speed and acceleration limit.

### 5.1 Architecture

The architecture of the ANN used for both models is composed by an input layer with 10 neurons (number of input variables), a hidden layer with 64 neurons, and an output layer with one neuron, which gives the energy consumption or power peak prediction, depending on the case. In this network, the ReLU (Rectified Linear Unit) activation function is used.

More sophisticated architectures were tested, such as nets with two or three hidden layers and ResNets. However, no meaningful advantages with respect to accuracy or efficiency were obtained, thus only the results with the aforementioned architecture are reported in this work.

### 5.2 Training

An amount of 1,450 data points (movements of the robotic arm) were used for the training of the model, that were divided into training, validation, and test sets as described in Table 6.

**TABLE 6:** Data partitioning.

Partition	Total percentage	Data quantity
Training	70%	1,014
Validation	15%	218
Test	15%	218

The optimization algorithm employed was the Adaptive Movement Estimation (Adam), with a learning rate set on 0.01. Mean Squared Error (MSE) was used as the cost function, commonly used to measure the performance of regression models. Table 7 summarizes the information of the ANN architecture and training parameters. The training was performed in Google Colab, using TensorFlow2 with standard sequential layers.

**TABLE 7:** Neural network architecture for the Energy consumption and Power peak models.

Parameter	Value
Input Layer	10
Hidden Layer	64
Output Layer	1
Activation Function	ReLU
Optimization Method	Adam
Learning Rate	0.01
Cost Function	MSE
Epochs	800

### 5.3 Validation

**FIGURE 6:**
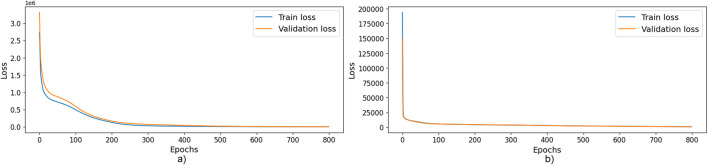
**(a)** Training and validation loss for the Energy consumption model. **(b)** Training and validation loss for the Power peak model.

According to the training loss curves, shown in Figure 6 for the training of both models, there is neither under-fitting nor over-fitting. After training, the mean absolute percentage error (MAPE) for the energy model was 0.0298 (i.e., an average absolute error of 2.98% ), and 0.0544 for the power peak model. The $R^{2}$ score was 0.998 for the energy model and 0.984 for the power peak model.

**FIGURE 7:**
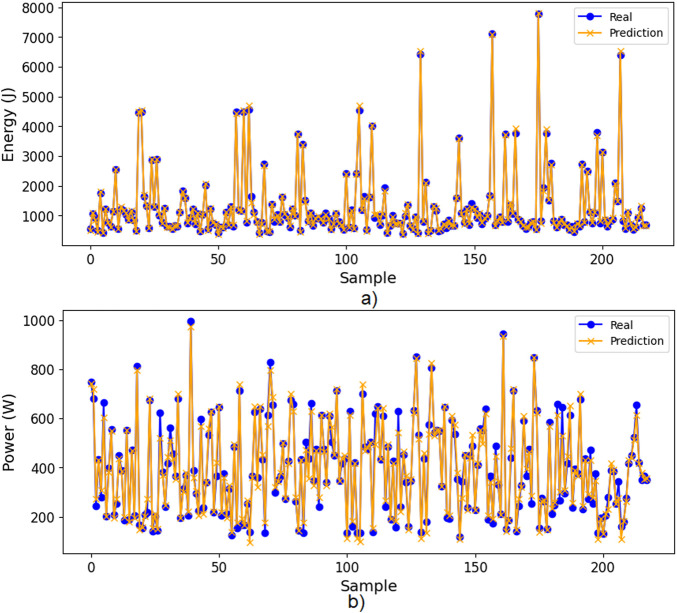
Comparison between real and predicted values from test data, **(a)** Energy model, **(b)** Power peak model.

Figures 7a,b show real and predicted values from the Test dataset for the Energy consumption model and the Power peak model, respectively. The resulting ANN models accurately predicted both the energy consumption per trial and the power peak, when the movement parameters are provided as input.

### 5.4 Example

The trained predictors can be used to select the best parameters before implementing the robot program. For instance, Table 8 shows the prediction of the energy consumed by performing the trajectory of Experiment 3 (i.e., moving from position P1 > P2 > … > P8) for the movement types MoveJ and MoveL, with speed 0.5  m/s and acceleration $1\;m/s^{2}$ for MoveL type, and speed 1rad/s and acceleration $1\;rad/s^{2}$ for MoveJ type. The last column of Table 8 shows the saved energy as percentage (w.r.t. the movement type with the largest energy consumption). By selecting the movement type with the lower energy consumption for each target position, the energy consumed after the complete trajectory is 12803J . The energy consumed if all the movements are MoveJ or MoveL are 13232J and 18037J , respectively. Thus, by selecting the best type for each target position, the energy consumed is reduced by 29.02% w.r.t. Using MoveL in all the movements, and 3.24% w.r.t. Using MoveJ in all the movements. In this way, the energy predictor allows to select the best parameters in each movement, obtaining an optimized robot program.

**TABLE 8:** Motion mode comparison.

Target position	MoveJ energy (J)	MoveL energy (J)	Best option	Saved energy (%)
P1	2,125	2,115	MoveL	0.45
P2	1,335	1,182	MoveL	11.50
P3	2,334	7,284	MoveJ	67.95
P4	1,621	1783	MoveJ	9.12
P5	1,198	1,159	MoveL	3.24
P6	1,348	1,164	MoveL	13.59
P7	1985	2,107	MoveJ	5.77
P8	1,283	1,239	MoveL	3.38

Both predictors described in this section are a practical solution, since the models exhibit one of the most basic ANN architectures: a sequential net with only one hidden layer, which can be easily implemented in any device.

## 6 Optimization

The models obtained in the previous section for energy and power peak consumption can be used to optimize the movement parameters, so the robot programmer can set the best parameters that produce the robot movement with the lowest energy consumption, but keeping the power peak in a convenient value.

### 6.1 Problem formulation and method

**FIGURE 8:**
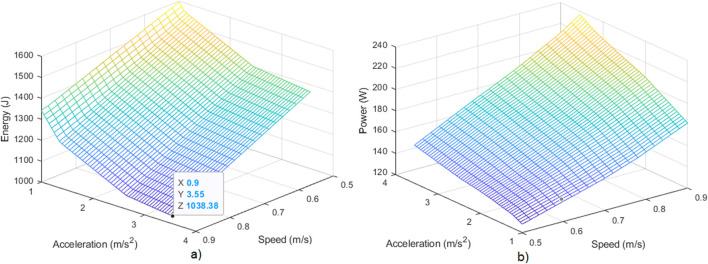
**(a)** Energy consumed for different speed and acceleration limit values. **(b)** Power peak consumed for the same values. The payload, initial and target coordinates are fixed.

Figure 8 shows the energy and power peak for different values of speed and acceleration limits, within the range (0.5  m/s,0.9m/s) and $\left(1\;m/s^{2},3.6\;m/s^{2}\right)$ , respectively, obtained with the ANN models. The payload is fixed as 0.3kg . The initial coordinates are those of position 6 in Table 3, and the target coordinates are those of position 7.

As it can be expected from previous discussion, given a fixed speed, the larger the acceleration limit, the lower the total energy and the higher the power peak. Similarly, for a given acceleration limit, the larger the speed, the lower the total energy and the higher the power peak. Thus, the question is to obtain the speed and acceleration limit parameters that provide the lowest energy consumption while keeping the power peak lower than a given bound.

**FIGURE 9:**
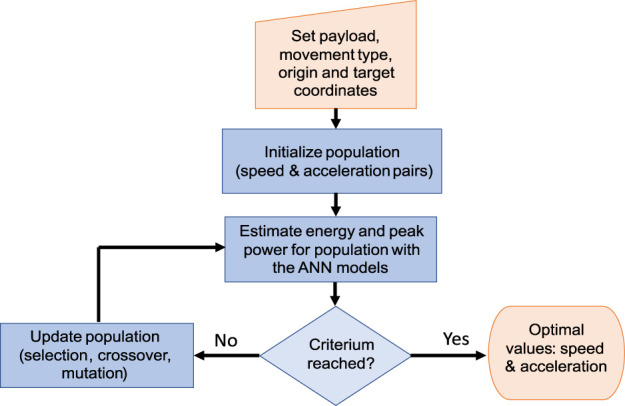
Flow diagram: genetic algorithm for calculating optimal speed and acceleration limits based on the ANN energy and peak power predictors.

For this, an optimization genetic algorithm is proposed, as described in Figure 9. In this, the population consists in pairs (speed limit, acceleration limit) within the allowable parameters range. Then, the consumed energy and power peak are calculated by the ANN predictors, for each member of the population. Classic genetic algorithm operators are used to update the population.

### 6.2 Example

**FIGURE 10:**
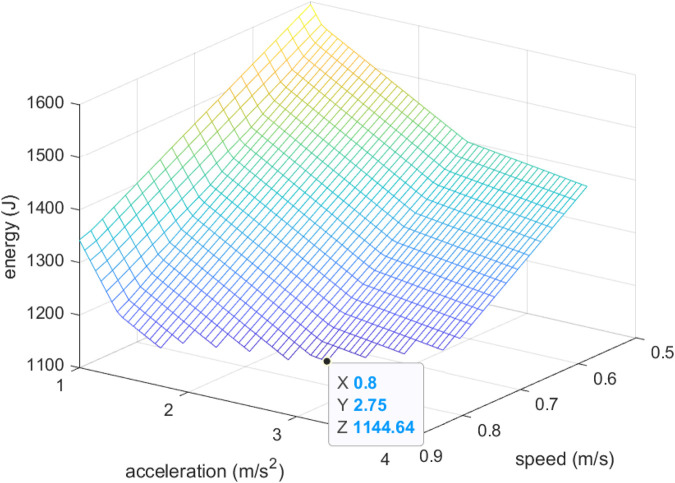
Energy consumed for different speed and acceleration limit values, for those cases where power peak is lower than 200W . The payload, initial and target coordinates are fixed.

The optimization algorithm was applied by using the multi-objective optimization library pymoo (Blank and Deb, 2020), considering speed and acceleration limits within ranges (0.5  m/s,0.9m/s) and $\left(1\;m/s^{2},3.6\;m/s^{2}\right)$ , respectively, a payload of 0.3kg , for moving the robot from position 6 to position 7, with a power peak bound of 200W . The population was set as 20, performing 10 generations (terminate criterion), using a random selection, polynomial mutation with η=1 , and simulated binary crossover with η=1 . Figure 10 shows the energy consumed, as in Figure 8a, but only showing the cases where the power is lower than 200W . The optimal speed and acceleration limits are calculated by the algorithm as v=0.8m/s and $a=2.756\;m/s^{2}$ , the consumed total energy is 1144.64J and the power peak is 200W , thus obtaining the lowest energy consumption at the edge of the peak power constraint. Notice from Figure 10 that there are combinations of limit-speed/limit-acceleration that produce similar energy consumptions (the lower points in the plot surface).

### 6.3 Optimization with intermediate points

Intermediate points are usually programmed in robot trajectories to avoid obstacles. Given an initial, an intermediate and a target position, the energy consumed per trajectory can be estimated as the sum of the energy spent from the initial to the intermediate position, and the energy spent from the intermediate to the target position. This will result in an overestimation, since the robot may not need to completely decelerate when approaching the intermediate position, and then to accelerate from the intermediate position. However, the difference in the energy consumption will be small for collaborative robots. For instance, considering the data of Experiment 1 with payload 5kg and acceleration $1.6\;m/s^{2}$ , the energy consumed during acceleration can be approximately computed as 61.2J , traveling a vertical distance of 0.128m . However, if the robot travels the same distance at the limit speed 0.7m/s (i.e., without accelerating), the energy spent would be approximately 29.25J , i.e., a difference of 31.95J . This represents a minimal percentage of the total trajectory energy consumption (take for reference the data in Figure 3, where energy per trajectory is above 1000J for the corresponding payload and acceleration limit). This small energy contribution due to acceleration is explained by the speed and acceleration limitations of collaborative robots, in comparison with the energy required to maintain the robot in a vertical pose, as discussed in Subsection 4.

Another question that may arise is if the energy consumption can be decreased by including intermediate points at certain positions to be determined. To address this question, the optimization algorithm of Figure 9 can be modified, by including the intermediate position coordinates as decision variables, and calculating the energy spent as the sum of the energy required from the initial position to the intermediate position, and the energy required from the intermediate position to the target position. This was tested for the previous case (subsection 6.2), considering a population of 50 and 20 generations (more iterations are required due to the additional variables). In this case, an intermediate point was calculated as 0.218m in X, 0.00m in Y, and 0.425m in Z; the obtained total energy with the intermediate point was 1404.1J , reaching a peak power of 199.2W . Thus, the obtained energy consumption is larger (in this case 256J larger) than that obtained without the intermediate point. Part of this increase in the energy consumption is due to the deceleration and acceleration energy required for the intermediate position. More experiments were conducted with other initial and target positions obtaining similar results: the inclusion of free intermediate positions do not improve the energy consumption.

## 7 Conclusion

This work presented an energy analysis for a collaborative robot UR10, paying particular attention to the total energy consumed and power peak in a movement trajectory, as a function of the initial and target coordinates, payload, movement type, maximum acceleration and maximum speed. Thanks to the capabilities offered by this robot model, the experimental data was collected by establishing a communication with the controller, without using any external sensor. Counterintuitively, it was found that the trajectory total consumed energy is lower for higher speed limits and higher acceleration limits. On the other hand, higher speed and acceleration limits produce higher power peaks. A couple of ANN models were trained to predict the power peak and the total energy consumed by the robot when performing a movement, under given parameters. The output of the models are the predicted energy consumption and the power peak, respectively, predicting these variables with high accuracy. These models are later used in an optimization genetic algorithm for obtaining the best speed and acceleration limit parameters that reduce the energy consumption while keeping the power peak under certain bound, for a given payload, initial and target coordinates.

The proposed methodology can be applied to obtain energy and power predictors for other cobot models, and then use them with the same optimization algorithm to obtain the best parameters. Be aware that predictors for one cobot model should not be extrapolated to another cobot model, since changes in geometry and weights may greatly affect the input-output variables relations due to the nonlinear nature of robots dynamic behaviors.

## Data Availability

The raw data supporting the conclusions of this article will be made available by the authors, without undue reservation.
